# Comprehensive Analyses of Stromal-Immune Score-Related Competing Endogenous RNA Networks In Colon Adenocarcinoma

**DOI:** 10.1155/2022/4235305

**Published:** 2022-05-14

**Authors:** Yalin Tong, Mengle Peng, Jinbei Li, Ying Niu

**Affiliations:** ^1^Department of Gastroenterology, The First Affiliated Hospital of Zhengzhou University, Zhengzhou, 450052 Henan, China; ^2^Department of Clinical Laboratory, Henan No. 3 Provincial People's Hospital, Zhengzhou, 450052 Henan, China; ^3^Department of Cardiology, The Second Affiliated Hospital of Zhengzhou University, Zhengzhou, 450052 Henan, China

## Abstract

Although recent clinical investigations emphasize the roles of myriad diversities of RNAs in stromal and immune components in the tumor microenvironment, especially in colon adenocarcinoma, however, analyses of “competing endogenous RNAs (ceRNA)” network in association with stromal and immune scores have yet to be determined. This study was conducted to explore the regulatory mechanisms of a stromal-immune score-based ceRNA network in colon adenocarcinoma. Stromal and immune scores of colon adenocarcinoma tumor samples were calculated by using the ESTIMATE algorithm. Differential expression analysis between samples with high/low stromal and immune scores was performed, followed by functional annotation for the overlapping DEmRNAs. The ceRNA network was constructed by differential expression analysis, prediction of RNA-RNA interaction, and correlation with clinicopathological parameters of the patients, which were further verified by external datasets and experiments. Colon adenocarcinoma patients having higher immune scores exhibited prolonged overall survival. RNA dataset analyses from TCGA revealed aberrant expressions of a total of 2052 mRNAs, 108 lncRNAs, and 70 miRNAs between high and low stromal/immune groups. Functional annotation mapped the differentially overexpressed mRNAs for immune-associated GO terms. To construct the ceRNA network, a total of 48 lncRNAs, 40 miRNAs, and 199 mRNAs were sorted out. A dysregulated ceRNA network consisting of 6 lncRNAs, 11 miRNAs, and 39 mRNAs was constructed by comparing RNA expressions between cancer as well as adjacent normal tissues. The ceRNA regulatory axis “MIAT/miR-532-3p/STC1” was regarded as a potential hit by the comprehensive analysis. The RT-qPCR assay showed upregulation of MIAT and STC1 while downregulation of hsa-miR-532-3p expression in cancer. Thus, our study highlights the potential role of a stromal-immune score-based ceRNA network in the colon adenocarcinoma microenvironment. The ceRNA axis MIAT/miR-532-3p/STC1 could serve as a promising therapeutic target for colon adenocarcinoma.

## 1. Introduction

Colon cancer (CC) is one of the most prevalent cancers worldwide and is considered the second leading cause of cancer-associated mortality [1]. Even though radical resection combined with chemotherapy serves as the main therapeutic strategy for colon cancer [2], however, owing to remarkable disease heterogeneity, the survival outcomes remain poor, and the 5-year overall survival rate is ~60% [3, 4], which urges for an investigation into novel therapeutic targets for colon cancer.

Noncoding transcripts such as long noncoding RNAs (lncRNAs), noncoding linear RNAs (miRNAs), and circular RNAs (circRNAs) have been shown to play vital roles in the onset of colon cancer [5]. The ceRNA networks are strongly associated with the progression of colon cancer [6]. Huang and Pan constructed a ceRNA-based model to predict the clinical prognosis of colon cancer [7]. Similarly, Wu et al. reported that the lncRNA MALAT1/miR-129-5p/HMGB1 axis initiates the development of the colon cancer [8]. ceRNA network analysis, thus, may help to figure out prospective genes involved in the early diagnosis and therapy of colon cancer.

Colon cancer tissues like other solid malignant tumor tissues consist of stromal cells, infiltrating immune cells, vascular cells, and other nontumor cells in addition to tumor cells [9]. Stromal and immune cells among others in the tumor microenvironment play critical roles in tumor formation and progression [10–13]. Furthermore, the stromal and immune cells are involved in antitumor immunoresponses in the colon cancer [14, 15]. Colon cancer datasets from TCGA database were used to construct a ceRNA network by using the ESTIMATE bioinformatic algorithm tool [16] to identify a set of biomarkers in the colon cancer tumor microenvironment. In the current study, we downloaded the colon cancer cohort's information from TCGA database, applied the ESTIMATE algorithm, and constructed the ceRNA networks to identify a set of biomarkers related to colon cancer's tumor microenvironment (TME).

## 2. Material and Methods

### 2.1. Data Processing for Stromal and Immune Scores

Gene expression data of the RNA and miRNA profiles as well as the corresponding clinical information of the colon cancer cohort were downloaded from TCGA database. Patients with more than one malignancies were excluded. Finally, the whole RNA sequence data of 480 colon adenocarcinoma samples were collected. This study was conducted by the publication guidelines provided by TCGA (http://cancergenome.nih.gov/publications/publicationguidelines). The GSE33113 and GSE18392 cohorts with normal colon adenocarcinoma samples were utilized to determine the expression levels of the candidate genes. The paired *t*-test was applied to normally distributed data while the Wilcoxon rank test was used in the identification of differentially expressed genes (DEGs) between normal and tumor tissues. The average mRNA expression level was obtained by matching multiple probes with one symbol. The fraction of immune and stromal cells in each tumor sample based on gene expression profiles was estimated by using ESTIMATE.

### 2.2. Relating Disease Prognosis with Stromal/Immune Scores

Tumor samples with complete survival records were used for survival analysis. According to the stromal and immune scores, the patients were divided into two groups with an optimal cutoff, which was identified by employing the R package “maxstat.” The R package “survminer” was subsequently applied to construct a survival curve, and the log-rank test was used to compare the survival outcomes.

### 2.3. DEGs Analysis between High and Low Immune Score/Stromal Score Groups

Based on the optimal cutoff of the immune and stromal scores, patients were divided into high-score groups and low-score groups. The Limma package of the R software was used to screen the differentially expressed lncRNAs, mRNAs, and miRNAs between the high and low immune/stromal-score groups according to the standard criterion (|*FC*| > 1.2, a *p* value < 0.05). We identified the differentially expressed lncRNAs, mRNAs, and miRNAs between the immune scores and stromal scores by Venn diagram intersections. Heatmaps and clustering were generated and performed, respectively, using the R package “heat map.”

### 2.4. GO (Gene Ontology) and KEGG (Kyoto Encyclopedia of Genes and Genome) Enrichment Analyses

GO and KEGG pathway enrichment analyses were performed using the “clusterProfiler,” “enrichplot,” and “ggplot2” packages in the R environment. Only those terms with both *p* and *q* values < 0.05 were considered to be significant.

### 2.5. Construction of a ceRNA Regulatory Network Involved in Colon Cancer

A ceRNA network was constructed using common differentially expressed miRNAs, lncRNAs, and mRNAs in patients. The miRanda (http://www.microrna.org/) and TargetScan (http://www.targetscan.org/) databases were used to identify lncRNA-miRNA interactions. The miRanda (http://www.microrna.org/) and PITA (https://genie.weizmann.ac.il/pubs/mir07/mir07exe.html) databases were used to predict miRNA-mRNA interactions. The correlations of miRNA-lncRNA pairs, as well as miRNA-mRNA pairs, were analyzed by Pearson's correlation analysis. Cytoscape (v3.6.0) was used to visualize the ceRNA network.

### 2.6. Protein-Protein Interaction (PPI) Network Construction

To explore the interaction among proteins in the ceRNA network, we constructed a PPI network using the STRING database. The Cytoscape 3.6.1 software was applied to visualize the PPI network. The larger nodes represent proteins with a higher degree.

### 2.7. Patients and Tumor Tissues

Tissues from human colon adenocarcinoma and matched adjacent specimens were collected from 20 patients who underwent colectomy. Colon adenocarcinoma was confirmed by histopathological examination. The Ethics Committee of the First Affiliated Hospital of Zhengzhou University has approved the sampling and experimental procedures, and informed written consent was obtained from all patients involved in the study.

### 2.8. RNA Extraction and qRT-PCR

According to the manufacturer's protocol, total RNAs were extracted using TRIzol reagent (Thermo Fisher Scientific, USA), and quality and quantity of the RNA samples were detected by using Nanodrop. For detection of hsa-miR-532-3p, the cDNA was synthesized using miRNA 1^st^ Strand cDNA Synthesis Kit (by stem-loop) (Vazyme, china). qRT-PCR was performed using miRNA Universal SYBR qPCR Master Mix (Vazyme, china). The lncRNA and mRNA cDNA was synthesized using TIANScript II RT Kit (Tiangen, China) and detected by FastFire qPCR PreMix (Tiangen, China) according to the manufacturer's instructions. GAPDH and U6 were used as internal references for the quantification of lncRNAs, mRNAs, and miRNAs, respectively. The 2^−*ΔΔ*Ct^ method was used to calculate the relative RNA expression. All primers were purchased from GENEWIZ of China. The primer sequences of qRT-PCR are as follows:

MIAT-F: 5′-GCACCTTGAGTGAATGTCAAGGCAG-3′, MIAT-R: 5′-TGGCAGCATCCAGCCGACACACAGG-3′; ARL4C-F: 5′-CCAGTCCCTGCATATCGTCAT-3′, ARL4C-R: 5′-TTCACGAACTCGTTGAACTTGA-3′; STC1-F: 5′-TTCACTCAAGCCAGGAGAGGGAAAG-3′, STC1-R: 5′-AGGCATGCAAAAGCCCCGCAG-3′; GAPDH-F: 5′-TGAACGGGAAGCTCACTGG-3′, GAPDH-R: 5′-TCCACCACCCTGTTGCTGTA-3′; hsa-miR-532-3p-F: 5′-GCCTCCCACACCCAAGG-3′, hsa-miR-532-3p-R: 5′-AGTGCAGGGTCCGAGGTATT-3′; and U6-F: 5′-CTCGCTTCGGCAGCACA-3′, U6-R: 5′-AACGCTTCACGAATTTGCGT-3′.

### 2.9. Statistical Analyses

The statistical analyses were performed by using GraphPad prism (V7.0) and R-packages (version 3.6.4). *p* < 0.05 was considered significant.

## 3. Results

### 3.1. RNA Sequencing, Data Mining, and Processing

**Figure 1:**
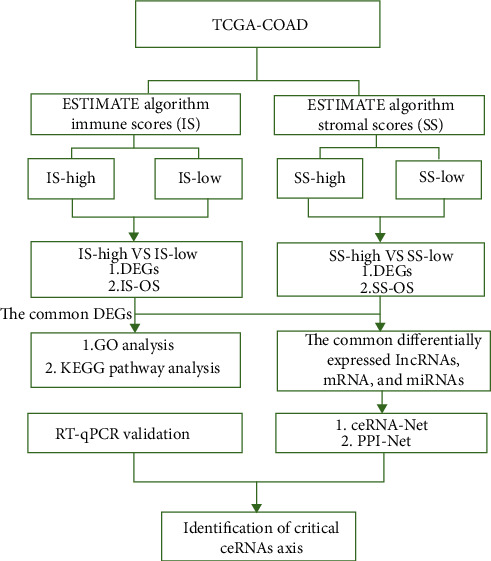
Workflow of the dataset processing.

The overall workflow of our study is shown in Figure 1. The RNA sequencing data were downloaded from TCGA database. After data preprocessing, we considered the data of 480 pure colon adenocarcinoma samples for further analyses. Immune scores and stromal scores were generated by the ESTIMATE algorithm; then, based on the immune and stromal scores, the patients were divided into different groups. The top differentially expressed genes in colon adenocarcinoma samples were further evaluated by KEGG and Gene Ontology enrichment analyses. Then, common differentially expressed lncRNAs, mRNAs, and miRNAs were used to construct a ceRNA network followed by PPI network construction based on mRNAs in the ceRNA network. The prognostic values of mRNAs in this ceRNA network were tested by the log-rank test. The differences in expression of lncRNAs, mRNAs, and miRNAs included in the ceRNA network were experimentally validated in clinical samples using external datasets.

### 3.2. Scores Were Correlated with the Survival of Colon Adenocarcinoma Patients

**Figure 2:**
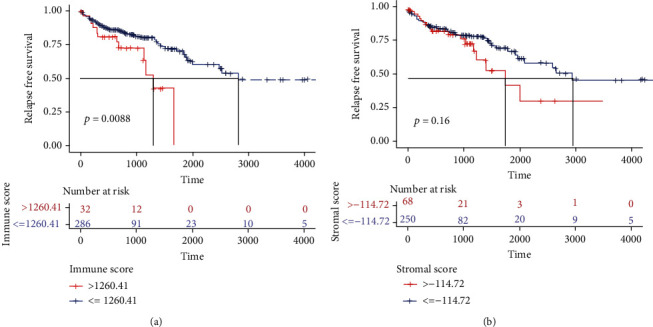
Scores were correlated with the survival of colon adenocarcinoma patients. (a) Kaplan–Meier survival analysis of overall survival for patients with low vs. high immune scores. (b) Kaplan–Meier survival analysis of overall survival for patients with low vs. high stromal scores.

The immune scores showed a significant difference among the high- and low-risk groups of colon adenocarcinoma patients. The patients in the high immune score group showed poor overall survival (*p* < 0.01, HR; 95% CI) (Figure 2(a)), and the patients with lower stromal scores showed longer overall survival, although it was not statistically significant (Figure 2(b)). These results implied that the immune cell percentage in colon cancer tissues is a more suitable indicator for the prognosis of colon cancer.

### 3.3. Differentially Expressed RNA Identification

**Figure 3:**
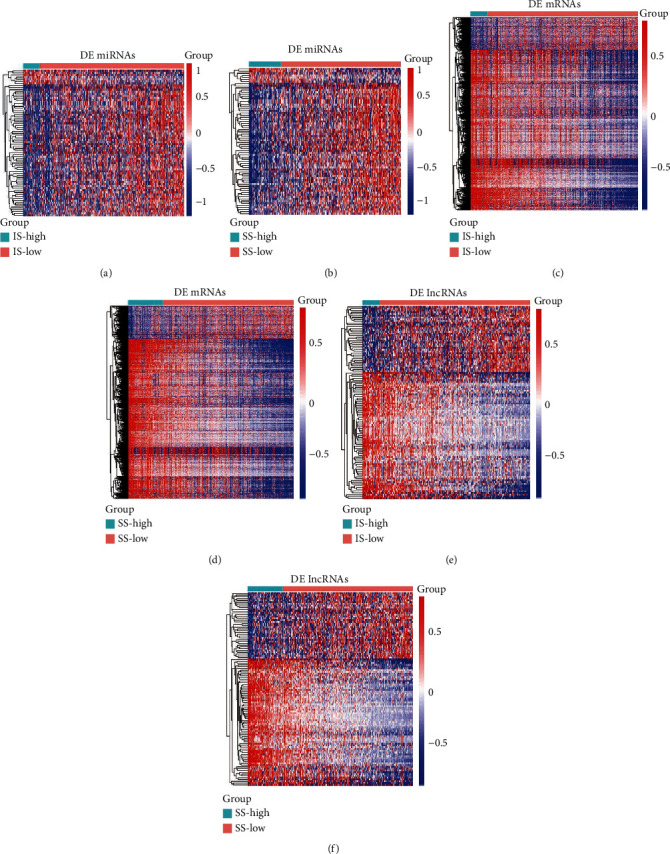
Expression profiles of stromal and immune score-related differentially expressed genes (DEGs). (a, c, e) Heatmap of differentially expressed miRNAs, mRNAs, and lncRNAs between the high and low immune groups. (b, d, f) Heatmap of differentially expressed miRNAs, mRNAs, and lncRNAs between the high and low stromal groups. The left vertical axis presents the DEG clusters. The horizontal axis represents the samples. The blue color represents downregulated genes, and the red color represents upregulated genes.

To identify the exact changes in RNA expression profiles in the tumor microenvironment, the comparison between high immune (or stromal) score and low immune (or stromal) score samples revealed immune-related DE RNAs including 3201 DE mRNAs, 327 DE lncRNAs, and 95 DE miRNAs. Among them, 2185 mRNAs, 152 lncRNAs, and 24 miRNAs were upregulated, and 1015 mRNAs, 174 lncRNAs, and 71 miRNAs were downregulated. At the same time, a total of 3849 DE mRNAs, 273 DE lncRNAs, and 309 DE miRNAs were identified as stromal-related DE RNAs. Among all, 2468 mRNAs, 143 lncRNAs, and 65 miRNAs were upregulated, and 1381 mRNAs, 130 lncRNAs, and 244 miRNAs were downregulated. The expression profiles of immune and stromal score-related DE miRNAs, mRNAs, and lncRNAs are presented as heatmaps (Figures 3(a)–3(f)). Finally, 70 shared miRNAs, 2052 mRNAs, and 108 lncRNAs between the immune and the stromal groups were filtered out for further analysis.

### 3.4. Functional Annotation of Differentially Expressed mRNA

**Figure 4:**
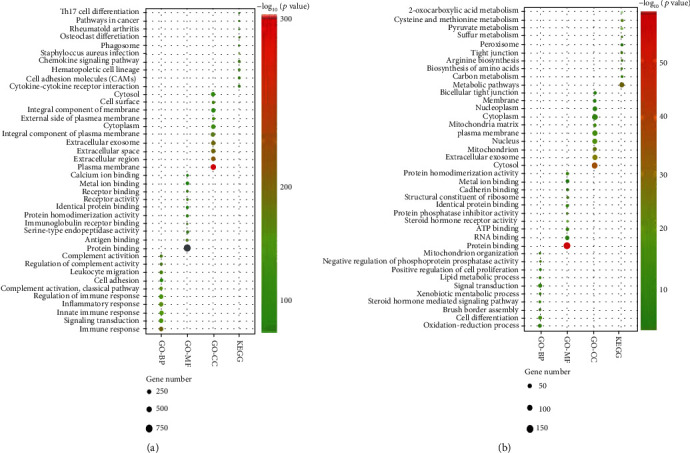
Enrichment analysis of common differentially expressed genes. (a) The top ten GO terms and KEGG pathways are enriched by the upregulated DEGs. (b) The top ten GO terms and KEGG pathways are enriched by the downregulated DEGs.

The GO functional enrichment analyses indicated that the shared overexpressed DEGs were nearly mainly involved in the immune response, antigen binding, and the innate immune response (Figure 4(a)). At the same time, the DEGs expressed at a low level showed association with cell migration, cell differentiation, and signal transduction (Figure 4(b)). KEGG enrichment analysis also displayed the enrichment of Th17 cell differentiation and chemokine signaling pathways for the overexpressed DEGs (Figure 4(a)), while the downregulated DEGs were mainly involved in some of the metabolic pathways (Figure 4(b)).

### 3.5. Construction of the ceRNA Regulatory Network of Colon Cancer

**Figure 5:**
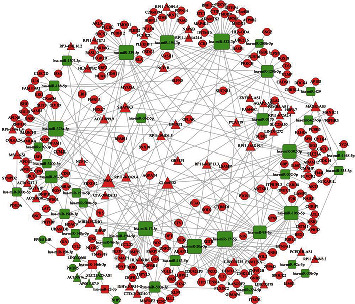
Competing endogenous RNA (ceRNA) networks. The rectangle, circle, and triangle in the figure represent microRNA, mRNA, and lncRNA, respectively. The red color represents upregulation in IS/SS-high, and the green color represents downregulation in IS/SS-high. The larger dot represents the stronger regulatory ability for mRNA.

By considering the interaction between dysregulated pairs of 146 miRNA-lncRNA pairs and 269 miRNA-mRNA, we constructed a lncRNA-miRNA-mRNA ceRNA network for colon cancer patients. In short, 47 lncRNAs, 39 miRNAs, and 198 mRNAs were filtered out of ceRNA networking. The final lncRNA-miRNA-mRNA ceRNA regulatory networks were prepared using Cytoscape (Figure 5).

### 3.6. PPI Network Construction

Based on the mRNAs in the ceRNA network, 227 pairs of protein interactions were identified by the STRING database. Using Cytoscape, 126 proteins were then chosen to establish a PPI network (supplement Figure 1) and the *CD86* showed the highest degree of interactions among others.

### 3.7. Identification of Potential Regulatory Axis

**Figure 6:**
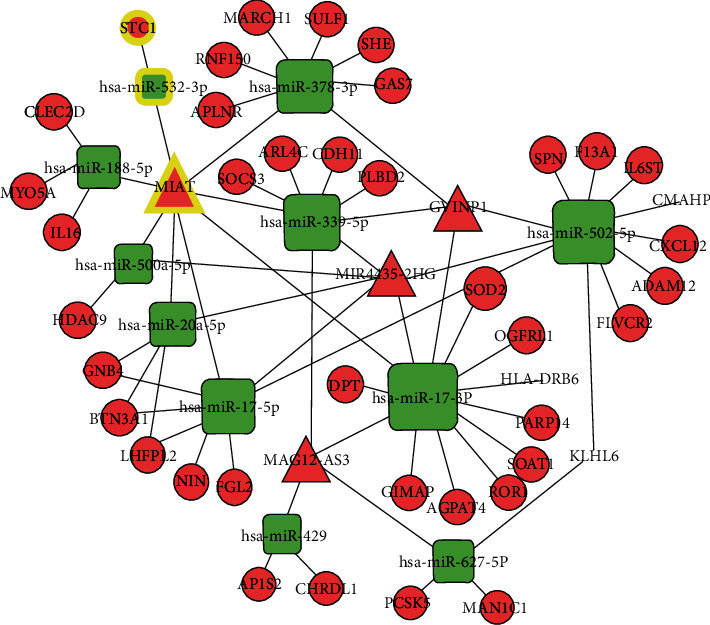
Dysregulated ceRNA network. The rectangle, circle, and triangle in the figure represent microRNA, mRNA, and lncRNA, respectively. The highlight color represents important interaction.

**Figure 7:**
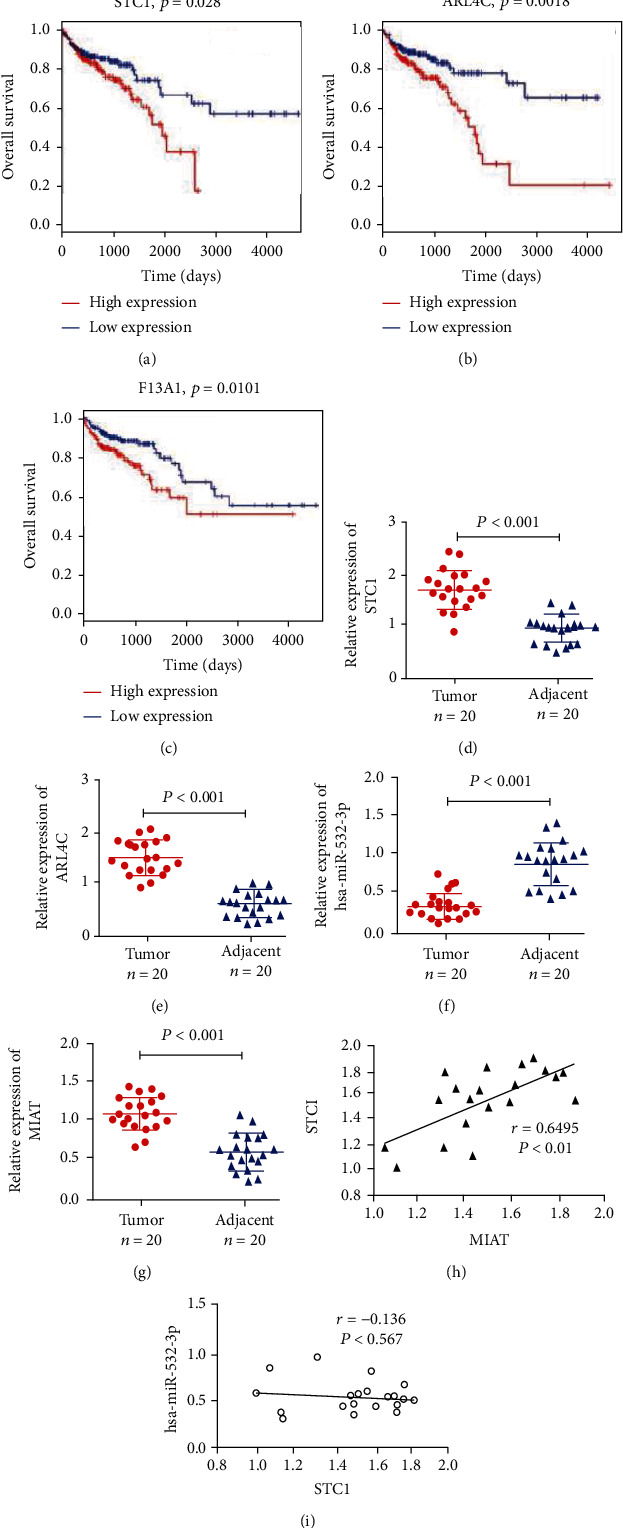
Identification of Potential Regulatory Axis. (a–c) Kaplan–Meier curve of mRNAs significantly correlated with overall survival. (d–g) Validation of the expression of representative RNAs by qRT-PCR in colon cancer tissues. (h, i) Correlation analysis between these predictive RNAs in colorectal cancer.

**Figure 8:**
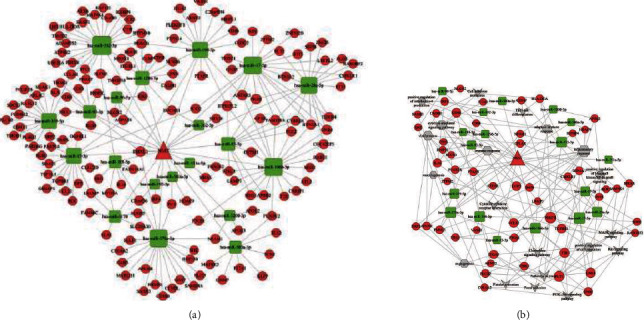
ceRNA network of MIAT. (a) The rectangle, circle, and triangle in the figure represent microRNA, mRNA, and lncRNA, respectively. (b) GO terms and KEGG pathways are enriched by the mRNA correlated with MIAT.

To identify the critical ceRNA axis that plays important role in biological processes, we screened DE RNAs between cancerous tissue and adjacent tissues by using GSE33113 and GSE18392 colon cancer cohorts. Finally, we identified 122 dysregulated ceRNA axis which included 6 lncRNA, 11 miRNA, and 39 mRNA (Figure 6). Among these mRNAs, 3 mRNAs (STC1, ARL4C, and F13A1) were related to the prognosis of colon adenocarcinoma patients (Figures 7(a)–7(c)). The relative expression of STC1 and ARL4C showed significant upregulation in colon tumor tissues (Figures 7(d) and 7(e)). Furthermore, we found ARL4C and STC1 were correlated with 4 lncRNA (GVINP1, MAGI2-AS3, MIAT, and MIR4435-2HG) and 2 miRNAs in the dysregulated ceRNA network. The evidence from the literature revealed that lncRNA MIAT plays a crucial role in cellular proliferation, migration, and invasion in various cancers. Moreover, only one miRNA was shown to have an association with a single gene (e.g., STC1) and a single lncRNA (e.g., MIAT); thus, we chose these miRNAs and lncRNA for ceRNA networking for further studies. The RT-qPCR assay showed that the expressions of MIAT were upregulated compared to colon cancer adjacent tissues (Figure 7(g)), while hsa-miR-532-3p was downregulated (Figure 7(f)). Furthermore, expression correlation analysis indicated a positive relationship between MIAT and STC1 (Figure 7(h)). However, no correlation between miRNA and mRNA as well as MIAT was observed (Figures 7(i)). To explore the potential mechanism of MIAT in colon adenocarcinoma, ceRNA networks of MIAT were constructed (Figure 8(a)). Functional annotation reveals that MIAT involves cytokine and chemokine signaling, immune response, cell adhesion, vasculogenesis, etc. (Figure 8(b)). Hence, our results revealed MIAT/miR-532/STC1 axis plays a crucial role in the occurrence, development, and prognosis of colon cancer.

## 4. Discussion

Recently, numerous studies have highlighted the influence of the tumor microenvironment on colon cancer initiation and development, indicating that the infiltration of immune cells and stromal cells could serve as promising sources for the novel prognostic and therapeutic biomarkers [9, 11, 17, 18]. Moreover, the role of immune-stromal-related genes in prognosis and therapy has attracted substantial attention in past years [19–21]; however, very few studies have investigated the ceRNA network associated with the TME of colon cancer.

In our study, we first found the association of high immune scores with longer overall survival in colon cancer patients which is consistent with a previous study showing that colon cancer patients with high immune levels have better clinical outcomes [19–22]. However, no correlation was observed between stromal score and overall survival in colon cancer patients. G. W. van Pelt et al. reported that the tumor-stroma ratio was an independent prognostic parameter in the colon cancer [23]. There may be differences exist between the findings of different methods applied for measuring the degree of immune cell infiltration in the tumor microenvironment. By comparing samples from TCGA with high or low stromal and immune scores, we identified 2052 mRNAs shared by patients of both groups. GO functional enrichment analyses found that the upregulated DEGs between high or low stromal and immune scores were mainly involved in some immune-related functions. The KEGG enrichment analysis displayed the enrichment of Th17 cell differentiation and the chemokine signal pathway in overexpressed DEGs. Indeed, Th17 cells can synthesize and secrete several types of cytokines and exert an important influence on oncogenesis and tumor progression in the colon cancer [24–26]. Chemokine signaling influences tumor immunity by recruiting bone marrow-derived MSC to the tumor microenvironment. Several chemokines such as CXCL1, CXCL12, and CXCL2 as well as CXCL17 were involved in the development, growth, and progression of the colon cancer [27–29]. These results show that these DEGs play an important role in the immune regulation process.

Dysregulated ceRNA network analysis implicated the involvements of CXCL12, IL16, HLA-DRB6, IL6ST, STC1, and BTN3A1 in immunity and inflammation regulation. Thus, this finding provides an insight into the association of the dysregulated ceRNA network with the onset and development of colorectal cancer by regulating the immune and inflammation progress. STC1 and ARL4C were found to be significantly upregulated in colon cancers. ARL4C was overexpressed in several cancer tissues with reported involvement in the initiation and progression of lung adenocarcinoma [30]. ARL4C promoted tumorigenesis in colon cancer, thereby representing a promising therapeutic target for curbing cancers [31]. ARL4C played an important role in glioblastoma and gastric cancer invasion and metastasis [32]. ARL4C is also suggested as a potential biomarker for poor prognosis in patients with renal cell carcinoma [33]. In pancreatic cancer, ARL4C participates in microenvironment remodeling and promotes tumor growth and drug resistance too [34]. Accumulating evidence had indicated that STC1 plays a crucial role in several different tumor types. Aberrant expression of STC1 had been observed in human carcinoma samples including colorectal cancer, breast cancer, lung adenocarcinoma, hepatocellular carcinoma, and thyroid carcinomas [35–37]. A recent study reveals that STC1 is a novel biomarker associated with immune characteristics and prognosis of bladder cancer [38] and also inhibits APC phagocytosis, contributes to tumor immune evasion and immunotherapy resistance [39]. In addition, STC1 can suppress the function of the macrophages [40]. In hepatocellular carcinoma, the upregulation of STC1 resulted in decreased energy metabolism [41]. STC1 regulates cellular apoptosis in cervical cancer via the NF-*κ*B pathway [42]. Another study demonstrated that STC1 can promote the invasion of breast cancer cells [43]. In colon cancer, the lncRNA-MALAT1/miR-101-3p/STC1 axis can promote the development of the tumor [44]. So, STC1 was regarded as the potential candidate for further analysis.

miR-532-3p is a highly conserved miRNA in many species. Accumulating evidence indicates that miR-532-3p serves as a tumor suppressor or promoter in multiple human cancers, such as gastric, renal, and liver cancers [45–47]. It was also reported that miR-532-3p regulated proinflammation of macrophages [48]. In addition, inflammation-dependent downregulation of miR-532-3p can contribute to the progress of the sarcopenia [49]. Recently, it has been shown that MIAT is widely overexpressed in many tumors, and the expression level of MIAT is positively correlated with lymph node metastasis, tumor stage, and prognosis of tumor patients [50]. As a tumor promoter, it regulates cell proliferation, migration, invasion, antiapoptosis, and other complex regulatory mechanisms [51–54], and MIAT-associated protein in our study also shows similar functions. Meanwhile, the role of lncRNA MIAT in immune cell infiltration has also been known, and lncRNA MIAT can serve as a biomarker for the prediction of immune cell infiltration in hepatocellular cancer and breast cancer [55, 56]. These results indicated that MIAT/miR-532-3p/STC1 could act as potential prognostic and diagnostic biomarkers.

## 5. Conclusions

In conclusion, a ceRNA network was constructed based on stromal-immune score-related DE RNAs. We also proposed an axis in which MIAT sponges miR-532-3p to regulate STC1. The expression of these RNAs included in the ceRNA network axis was validated in clinical samples. Our research provides novel insights that will improve the understanding of the stromal-immune score-related ceRNA network of colon cancer. However, there are some limitations of our study such as no detailed mechanisms of ceRNAs were performed experimentally, and the clinical use of these ceRNAs requires further investigation.

## Data Availability

The datasets analyzed for this study can be found in TCGA databases (https://portal.gdc.cancer.gov/repository) and the GEO databases (https://www.ncbi.nlm.nih.gov/geo/).

## Supplementary Materials

Supplementary Materials Supplement Figure 1: protein-protein interaction networks. Protein-protein interaction network of mRNA is included in the ceRNA. The red hexagons represent upregulated genes, and the green nodes represent downregulated genes.
