# Supplementary material for: Comprehensive Analyses of Stromal-Immune Score-Related Competing Endogenous RNA Networks In Colon Adenocarcinoma
Source: Dis Markers. 2022 May 14;2022:4235305. doi: 10.1155/2022/4235305 (PMC9124109; doi:10.1155/2022/4235305)
Supplement: Supplementary Materials — Supplement Figure 1: protein-protein interaction networks. Protein-protein interaction network of mRNA is included in the ceRNA. The red hexagons represent upregulated genes, and the green nodes represent downregulated genes. [file 4235305.f1.pdf]

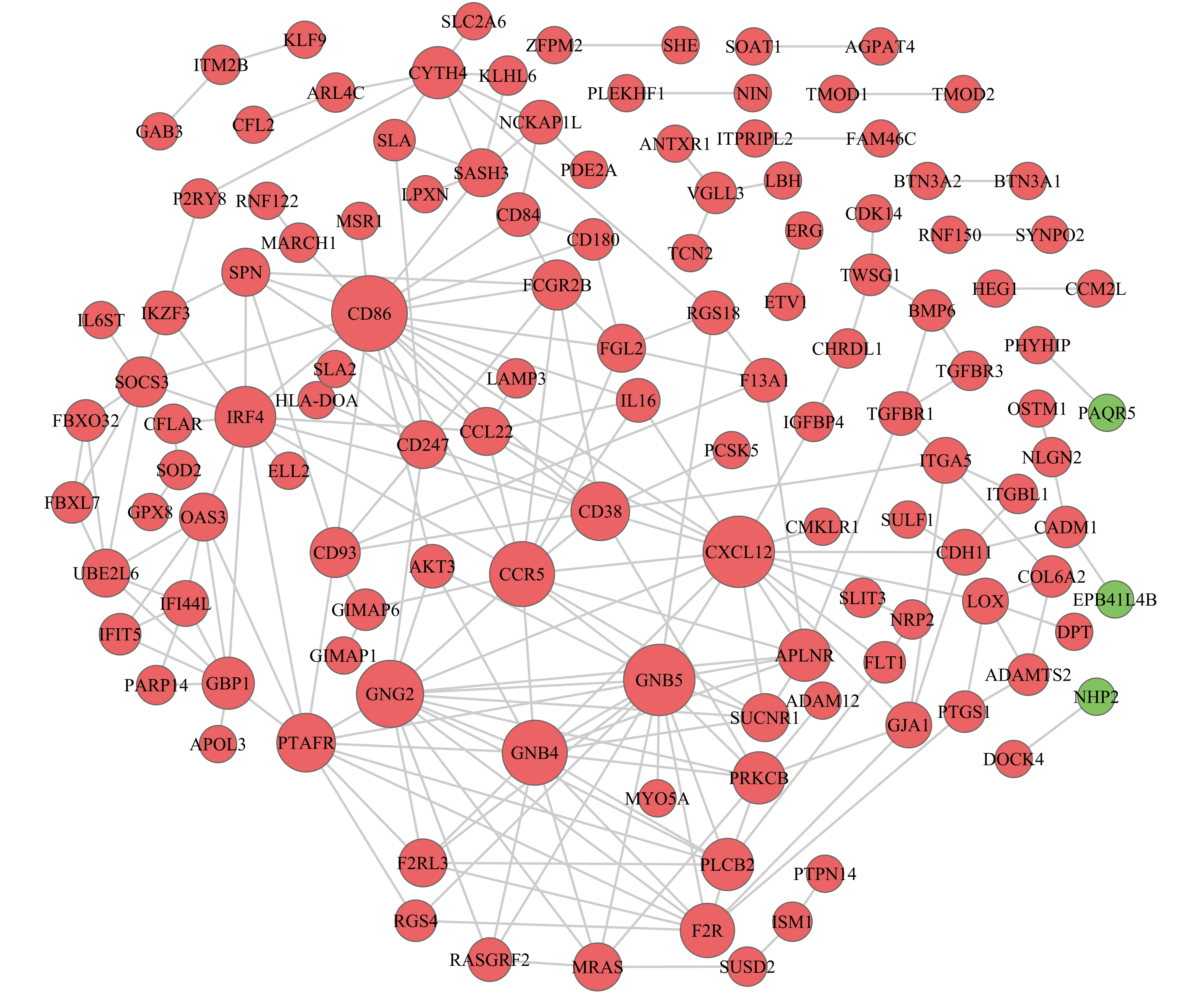
SLC2A6
KLF9
ZFPM2
SHE
SOAT1
AGPAT4
ITM2B
CYTH4
KLHL6
PLEKHF1
NIN
TMOD1
TMOD2
ARL4C
GAB3
CFL2
NCKAP1L
ANTXR1
ITPRIPL2
FAM46C
SLA
PDE2A
SASH3
LBH
BTN3A2
BTN3A1
P2RY8
RNF122
LPXN
VGLL3
MSR1
CD84
CDK14
RNF150
SYNPO2
MARCH1
CD180
ERG
TCN2
SPN
FCGR2B
TWSG1
HEG1
CCM2L
ETV1
IL6ST
IKZF3
CD86
RGS18
BMP6
FGL2
CHRDL1
PHYHIP
SOCS3
SLA2
LAMP3
F13A1
TGFBR3
HLA-DOA
IL16
OSTM1
PAQR5
IRF4
TGFBR1
FBXO32
CFLAR
CCL22
IGFBP4
CD247
PCSK5
ITGA5
NLGN2
SOD2
ELL2
FBXL7
GPX8
OAS3
CD38
ITGBL1
CMKLR1
SULF1
CADM1
CD93
CXCL12
CDH11
AKT3
CCR5
COL6A2
UBE2L6
SLIT3
LOX
EPB41L4B
IFI44L
GIMAP6
NRP2
DPT
IFIT5
APLNR
FLT1
GIMAP1
GNB5
ADAMTS2
NHP2
PARP14
GBP1
GNG2
ADAM12
SUCNR1
PTGS1
GJA1
APOL3
PTAFR
GNB4
PRKCB
DOCK4
MYO5A
F2RL3
PLCB2
PTPN14
RGS4
ISM1
F2R
RASGRF2
MRAS
SUSD2
